# Reduced Olfactory Bulb Volume in Obesity and Its Relation to Metabolic Health Status

**DOI:** 10.3389/fnhum.2020.586998

**Published:** 2020-11-27

**Authors:** Maria Poessel, Nora Breuer, Akshita Joshi, André Pampel, Arno Villringer, Thomas Hummel, Annette Horstmann

**Affiliations:** ^1^Department of Neurology, Max Planck Institute for Human Cognitive and Brain Sciences, Leipzig, Germany; ^2^Integriertes Forschungs- und Behandlungszentrum (IFB) Adiposity Diseases, Leipzig University Medical Center, Leipzig, Germany; ^3^Smell and Taste Clinic, Department of Otorhinolaryngology, University of Dresden Medical School, Dresden, Germany; ^4^Department of Neurophysics, Max Planck Institute for Human Cognitive and Brain Sciences, Leipzig, Germany; ^5^Day Clinic for Cognitive Neurology, University Hospital at the University of Leipzig, Leipzig, Germany; ^6^Berlin School of Mind and Brain, Mind Brain Body Institute, Humboldt-Universität zu Berlin, Berlin, German; ^7^Charité – Universitätsmedizin Berlin, Berlin, Germany; ^8^International Max Planck Research School on the Life Course, Max Planck Institute for Human Development, Berlin, Germany; ^9^International Max Planck Research School on the Neuroscience of Communication, Max Planck Institute for Human Cognitive and Brain Sciences, Leipzig, Germany; ^10^Department of Psychology and Logopedics, Faculty of Medicine, University of Helsinki, Helsinki, Finland; ^11^Leipzig University Medical Center, Collaborative Research Council (CRC) 1052A5 ‘Obesity Mechanisms’, Leipzig, Germany

**Keywords:** olfactory bulb, obesity, olfaction, smell perception, HOMA-IR, metabolic health

## Abstract

Smell perception plays an important role in eating behavior and might be involved in body weight gain. Since a body of literature implies that olfactory perception and function is hampered in obesity, we here investigate neuroanatomical correlates of this phenomenon. We assessed olfactory bulb (OB) volume with magnetic resonance imaging in 67 healthy participants with a body mass index (BMI) from 18.9 to 45.4 kg/m^2^ (mean = 28.58 ± 6.64). Moreover, we obtained psychophysiological data on olfactory ability (Sniffin’ Sticks, Food associated odor test) and self-report measurements on eating behavior. Additionally, we collected parameters associated with metabolic health in obesity (waist-hip ratio, waist-height ratio, leptin levels, body fat percentage, fat mass index, insulin resistance) to investigate recently proposed mechanistic explanatory models of why olfaction may be altered in obesity. We showed that OB volume was significantly lower in participants with obesity when compared to those of normal weight. Moreover, we found weak to moderate negative correlations between OB volume and BMI and related measures of metabolic health, especially leptin, body fat percentage, waist-height ratio and insulin resistance. However, neither OB volume nor BMI were related to olfactory function in our young and healthy sample. Nevertheless, our results provide first indications that obesity is associated with brain anatomical changes in the OBs.

## Introduction

Obesity has become a worldwide epidemic with an increased risk for major individual health consequences and a severe burden to the healthcare system (Stevens et al., 2012). Since obesity is a risk factor for many diseases such as diabetes type 2, cardiovascular diseases, certain forms of cancer and stroke (Rosenthal et al., 2017), it is essential to understand the factors that accompany excessive accumulation of body weight and its maintenance. One major factor driving the rapid increase of obesity is our obesogenic environment that encourages overconsumption of energy-dense foods even without physiological needs (Berthoud, 2012; Lopez-Gonzalez et al., 2020). Especially external cues, such as the smell of foods, can trigger appetite and intensify food cravings (Firmin et al., 2016). Thus, the olfactory system came into focus as an important contributor to unintentional weight gain.

In recent years, it has been shown that smell perception plays a significant role in the enjoyment of food and the control of food intake (Ramaekers et al., 2014, 2016; Proserpio et al., 2017). More specifically, olfaction influences food selection and meal size (Gaillet-Torrent et al., 2014) and triggers cephalic phase responses and cravings (Kitamura et al., 2010; Firmin et al., 2016; Proserpio et al., 2017). Hence, changes in olfactory perception might be involved in unhealthy eating and potentially lead to weight gain. On this notion, it has been shown that individuals with obesity show several alterations in the olfactory system. First, they have a higher hedonic response to palatable food odors when compared to people of normal weight (Stafford and Whittle, 2015). Second, it is widely accepted that individuals with obesity have decreased olfactory function (Richardson et al., 2004; Skrandies and Zschieschang, 2015; Fernández-Aranda et al., 2016; Fernandez-Garcia et al., 2017; Peng et al., 2019). Especially odor sensitivity, which reflects perceptual function (Hedner et al., 2010), is decreased in individuals with obesity when compared to people with normal weight (Skrandies and Zschieschang, 2015; Fernandez-Garcia et al., 2017).

The mechanisms behind these alterations remain unclear, however, it has been suggested that decreased olfactory function may be caused by hormonal and metabolic changes that are associated with obesity (Peng et al., 2019). This is supported by a recent finding of our working group, where we could show that high insulin resistance has a negative effect on food odor sensitivity in obesity (Poessel et al., 2020). Moreover, these hormonal changes might lead to altered function of the olfactory bulbs (Lacroix et al., 2015). Notably, the OBs have a high density of insulin and leptin receptors (Havrankova et al., 1981; Baskin et al., 1983; Marks et al., 1990; Thanarajah et al., 2019), hormones that are elevated in obesity and involved in homeostatic signaling (Murphy and Bloom, 2006; Durham, 2016; Lean and Malkova, 2016) as well as in modulating odor sensitivity (Tong et al., 2011; Brunner et al., 2013).

Interestingly, not all individuals with obesity show significant alterations in their metabolic and endocrine systems, referring to the concept of metabolically “healthy” obesity (Blüher, 2020). Those individuals are characterized by preserved insulin sensitivity and beta cell function, better cardiorespiratory fitness and lower visceral fat. In this respect, we believe that the olfactory system of metabolically healthy individuals with obesity might be less affected by these alterations. Consequently, we not only focus on body mass index (BMI) criteria for weight status, but additionally examine waist-height ratio (WHtR), waist-hip ratio (WHR), and leptin as proxies of body fat (Münzberg and Heymsfield, 2015; Christen et al., 2018; Landecho et al., 2019) and insulin resistance as assessed by homeostatic model assessment (HOMA-IR) (Gutch et al., 2015) and total body fat percentage as well as fat mass index (FMI).

The olfactory performance in standard tests (identification, discrimination and threshold tests) is reflected in the size of the olfactory bulb (OB) in participants with and without olfactory dysfunction (Buschhüter et al., 2008; Mazal et al., 2016). OB volume is reduced in various diseases that are associated with low olfactory performance, such as Parkinson’s disease, Alzheimer’s disease, schizophrenia, and depression (Turetsky et al., 2000; Thomann et al., 2009; Negoias et al., 2010; Liu et al., 2017). However, olfactory function was in most, but not in all cases negatively related to OB volume (Turetsky et al., 2000; Schriever et al., 2013). It is discussed that an insufficient afferent input of olfactory information from the olfactory receptor neurons to the OB causes the reduction in bulb volume (Gudziol et al., 2009). In addition, animal studies have reliably demonstrated that obesity leads to structural and functional changes in the olfactory system (Fadool et al., 2011; Thiebaud et al., 2014; Rivière et al., 2016). Since brain anatomical correlations to altered smell perception in human obesity have not been studied yet, we here investigate whether OB volume is associated with BMI and associated metabolic and hormonal markers of obesity, such as WHR, WHtR, body fat percentage, FMI, plasma level of leptin and insulin resistance. Further, we assessed olfactory function with the Sniffin’ Sticks test battery. Moreover, we explore whether eating behavior as assessed by subjective reporting via questionnaires correlates with brain anatomical changes in the olfactory primary pathways.

## Materials and Methods

### Participants

The sample consisted of 67 participants (33 women, 34 men), 28 participants had normal weight (BMI = 18.5 − 24.9 kg/m^2^), 28 participants were obese (BMI > 30 kg/m^2^), and 11 participants were overweight (BMI = 25 − 29.9 kg/m^2^) (see Table 1 for details). All participants were recruited from the Max Planck Institute for Human Cognitive and Brain Sciences database in Leipzig, Germany. They were aged between 21 and 41 years (28.5 ± 4.6 years) to minimize the impact of age on olfactory performance (Hummel et al., 2007). We excluded current or recent smokers (<3 years of abstinence) and subjects with allergies, history of nose surgery (except childhood adenoidectomy) and metabolic diseases (e.g., thyroid diseases or diabetes mellitus). Further exclusion criteria were vegetarian/vegan diet, history of neurological or psychiatric disorders, current use of medication (except oral contraceptives), drug use within the last 4 weeks and alcoholism. Pregnant and currently breastfeeding women were excluded for ethical reasons and because smell perception is altered in these conditions. All participants were previously screened by means of telephone interviews and had to meet our inclusion criteria (age between 18 and 45 years). After inclusion, participants provided written informed consent. The study was carried out in accordance with the Declaration of Helsinki and was approved by the Ethics Committee of the University of Leipzig (Reference number 387/17-ek, date of vote: 2017-10-17).

**TABLE 1:** Sample characteristics.

	Total	Normal weight	Obese		Overweight
		
	Mean ± SD (range)			*p*-value/*F*-value between NW and OBE	
**Characteristics**					
n	67	28	28		11
Sex	33 ♀, 34 ♂	14 ♀, 14 ♂	14 ♀, 14 ♂		5 ♀, 6 ♂
Age (years)	28.5 ± 4.6 (21 − 41)	27.1 ± 4.3 (21 − 35)	29.5 ± 5.1 (21 − 41)	0.109/2.3^*a*^	29.5 ± 3.0
BMI (kg/m^2^)	28.4 ± 6.6 (18.9 − 45.4)	22.3 ± 1.6 (18.9 − 24.9)	35.1 ± 4.3 (30.1 − 45.4)	<0.001***/212.834^*a*^	26.9 ± 1.4
WHR	0.89 ± 0.08 (0.75 − 1.05)	0.84 ± 0.06 (0.75 − 0.98)	0.93 ± 0.08 (0.80 − 1.05)	<0.001***/23.875^*b*^	0.88 ± 0.06
WHtR	0.52 ± 0.09 (0.38 − 0.71)	0.43 ± 0.04 (0.38 − 0.53)	0.61 ± 0.05 (0.51 − 0.71)	<0.001***/222.22^*b*^	0.51 ± 0.03 (0.45 − 0.55)
Body fat percentage (%)	28.43 ± 11.60 (8.21 − 60.60)	20.56 ± 6.02 (8.21 − 30.51)	37.50 ± 10.65 (11.99 − 60.60)	<0.001***/53.658^*b*^	25.39 ± 8.31
FMI	0.15 ± 0.09 (0.03 − 0.43)	0.08 ± 0.02 (0.03 − 0.11)	0.23 ± 0.08 (0.07 − 0.43)	<0.001***/92.03^*b*^	0.12 ± 0.50 (0.07 − 0.18)
BDI sum score	3.0 ± 3.3 (0 − 13)	2.0 ± 2.8 (0 − 11)	4.2 ± 3.8 (0 − 13)	0.018*/5.905^*a*^	2.5 ± 2.5
Passive smoking hours	1.5 ± 2.7 (0 − 15)	1.5 ± 2.9 (0 − 15)	1.5 ± 3.0 (0 − 15)	0.964/0.002^*a*^	1.5 ± 1.5
**Hormonal profile**					
HOMA-IR	1.14 ± 0.91 (0.10 − 5.10)	0.69 ± 0.38 (0.10 − 1.80)	1.73 ± 1.10 (0.60 − 5.10)	<0.001***/21.903^*a*^	0.78 ± 0.37
Leptin (ng/ml)	15.41 ± 13.91 (0.10 − 61.90)	8.13 ± 6.23 (0.10 − 27.70)	24.04 ± 15.95 (3.40 − 61.90)	<0.001***/21.878^*b*^	9.97 ± 8.04
Insulin (pmol/l)	60.18 ± 49.36 (7.00 − 278.30)	36.36 ± 19.82 (7.00 − 92.60)	91.68 ± 60.37 (30.40 − 278.30)	<0.001***/21.222^*a*^	40.59 ± 19.74
Glucose (mmol/l)	5.32 ± 0.43 (4.58 − 6.45)	5.15 ± 0.45 (4.58 − 6.16)	5.54 ± 0.39 (4.62 − 6.45)	0.001**/11.777^*a*^	5.18 ± 0.18

Data are presented as mean values, standard deviations, and minimum and maximum values. *^*a*^One-way analysis of variance (ANOVA). ^*b*^One-way analysis of variance with the covariate sex (ANCOVA). BMI, body mass index; WHR, waist-hip ratio; WHtR, waist-height ratio; FMI, fat mass index; BDI sum score, sum score of Beck’s Depression inventory; HOMA-IR, homeostatic model assessment of insulin resistance.*^∗^*p*≤0.05; ^∗∗^*p*≤0.01; ^***^p≤0.001.

### Study Design

The data presented here are part of a larger, multi-centered study in Saxony, Germany. We here only present the data of participants that were tested in one location at the Max Planck Institute for Human Cognitive and Brain Sciences in Leipzig. Participants were tested on 2 consecutive days. On the first test day, we collected a blood sample after an overnight fasting period of approximately 12 h. All participants were screened for normal olfactory function with the short form of the Sniffin’ Sticks odor identification test (Mueller and Renner, 2006). They further underwent a medical examination to assess body weight, height, waist and hip circumference and body fat percentage (measured by body impedance analysis). We conducted several questionnaires and interviews to assess eating behavior, past and present smoking behavior, including passive smoking, as well as information about the menstrual cycle phase of female participants. Moreover, we conducted a computer-based behavioral task and saliva tests on the first test day that are not part of the present data presentation in this manuscript. On the second test day, participants underwent after a 2 h fasting period a 50-min MRI scan, consisting of an anatomical and a functional part. After scanning, participants completed another set of questionnaires. In this manuscript, we only focus on the anatomical scan, the fMRI experiment is part of another subproject.

### Materials

#### Olfactory Tests

Olfactory testing was performed with the commercially available Sniffin’ Sticks^®^ test battery (Sniffin’ Sticks; Burghart Instruments, Wedel, Germany). It is a well-validated instrument to assess olfactory performance in the clinical and research context (Hummel et al., 1997). It includes subtests for odor threshold, odor discrimination and odor identification. In the threshold test, 16 triplets of felt-tip like pens are presented to the participant (Kobal et al., 1996; Hummel et al., 1997). In each triplet, one pen contains n-butanol, diluted in aqua conservans and concentrated from 1.22 ppm in pen number 16 up to 4% in pen number 1, whereas the other two pens contain only the solvent and serve as blanks. The odorized pen of each triplet must be identified correctly, starting with the lowest concentration. Participants are blindfolded to avoid visual identification. In a single staircase procedure, a higher concentration is presented following an incorrect choice, and a lower concentration following two correct identifications in a row. This procedure is repeated until seven staircase reversals are reached. The threshold is defined as the mean of the four last reversals of the staircase. A higher score signals higher capacity to pick up odors from the environment. The odor discrimination test assesses the participant’s ability to discriminate between different odorants. The task is to identify from three pens which one smells different than the other two pens. The odor identification test assesses the participant’s ability to correctly identify commonly known odors from a list of four descriptors for each odorized pen. The odors are similar in intensity and include: orange, peppermint, turpentine, cloves, leather, banana, garlic, rose, fish, lemon, coffee, anise, cinnamon, liquorice, apple, pineapple. In each test the sum of correct answers can range from 0 to 16. The sum of all three subtests results in the composite TDI-score and reflects general olfactory capacity, it can range from 0 to 48. Hereby, a TDI score of ≥30.5 is defined as normosmia (=normal smell function), a score between 16.5 and 30 hyposmia (=reduced smell function) and ≤16.5 anosmia (=functional smell impairment) (Hummel et al., 2007). Additionally, we obtained odor identification data from a newly developed food associated odor test (FAOT), here the participants had to identify the correct odor from a list of four descriptors. The test was developed to examine how naturalistic food odors are perceived. It includes the following odors: cinnamon, vanilla, coconut, bell pepper, caraway, peppermint, marzipan, butter, peach, liquorice, grape juice, cacao, cooked beef, bread, chicken, and fish (Denzer-Lippmann et al., 2017). Questionnaires and Interviews.

We initially screened for inclusion and exclusion criteria and education, using a self-developed screening questionnaire assessing smoking and drinking behavior, health status and subjective olfactory function. Depressive symptoms were assessed with the Beck Depression Inventory (BDI) (Beck et al., 1961) in a paper pencil form at the beginning of the first test day to control for acute suicidal tendencies and exclude participants with a sum-score >18 since depression is a confounding factor for smell impairment (Negoias et al., 2010). Additionally, participants were face to face interviewed about their smoking status by means of a smoking interview that was previously implemented in the Leipzig LIFE study (Loeffler et al., 2015). The interview contains questions about their past and present smoking behavior as well as passive smoking. Eating behavior was assessed by means of the German version (Nagl et al., 2016) of the Three factor Eating Questionnaire (TFEQ) (Stunkard and Messick, 1985). The TFEQ describes three dimensions of human eating behavior: cognitive restraint of eating, disinhibition and hunger. Dietary Fat and Free Sugar Short Questionnaire (DFS) (Fromm and Horstmann, 2019) that obtains data about monthly intake of saturated fat and free sugar within the last year. Moreover, we used the German versions of the Food Craving Questionnaires for food (FCQ) (Meule et al., 2014) and chocolate (FCQ-C) (Meule and Hormes, 2015). Both scales obtain information about experienced food cravings. The FCQ collects information about general state and trait cravings for food while the FCQ-C assesses craving and hunger for chocolate specifically. Furthermore, we obtained information on the menstrual cycle of women to assess cycle phase, since odor sensitivity is higher in follicular phase of the cycle / under oral contraception and lower in the luteal phase (Derntl et al., 2013; McNeil et al., 2013).

#### Blood Collection

Venous blood samples were collected after a fasting period of approximately 12 h, using 5.5 ml Sarstedt S-Monovette containers prepared with clotting activator for serum leptin and insulin and 2.7 ml Fluoride/EDTA preparations for glucose. Glucose tubes were immediately centrifuged at 3500 r.p.m. at 10°C and serum after standing at room temperature for 30 min. Both, serum and glucose were separated in two 1 ml tubes (Eppendorf Safe-Lock Tubes) and immediately after centrifugation frozen at -80°C.

### Neuroimaging

Brain imaging data were acquired using a 3 Tesla Siemens SKYRA scanner equipped with a 20-channel head coil. An MPRAGE (Mugler and Brookeman, 1990) dataset was acquired to create a T1-weighted (T1w) image. *TR* = 2,300 ms, echo time; *TE* = 2.98 ms, inversion times; *TI* = 900 ms (non-selective inversion recovery), flip angle; *FA* = 9°, nominal resolution = 1 mm isotropic. Right and left OB volume were determined using multislice T2-weighted turbo spin-echo images, with *TR*/*TE*/*FA* = 6,630 ms/126 ms/160°, acquired spatial resolution = 0.5 × 0.5 (in-plane), 1 mm slice thickness, 30 slices (no slice gap), and signal averages = 2.

### Data Analysis

R version 3.4.3. within RStudio (RStudio Team, 2016) was used for statistical evaluation. We used BMI as a continuous variable or as a grouping variable (normal weight: BMI 18.4 − 24.99 kg/m^2^, obese: BMI > 30 kg/m^2^). We used WHR, WHtR, circulating leptin levels, body fat percentage and FMI as additional measures of weight status, that are more related to metabolic health. Especially, WHtR and FMI have been identified as reliable predictors of metabolic risk in obesity (Łopatyński et al., 2003; Liu et al., 2013). Homeostasis Model Assessment of insulin resistance (HOMA-IR score) (Matthews et al., 1985), an index that serves as a proxy of insulin resistance, was then calculated from insulin and glucose by means of the HOMA2 Calculator^1^, applying the formula: HOMA-IR = glucose [mmol/l] × insulin [pmol/l]/135.

The α-level was set at 0.05. Bonferroni correction was applied to adjust the α-level for multiple testing. Whenever statistical assumptions for parametric testing were violated, we applied non-parametric robust tests. We defined outliers as values below or above 2.2 interquartile range from the samples lower or upper quartile (Hoaglin and Iglewicz, 1987).

Olfactory bulb size was assessed with 3D Slicer software (Fedorov et al., 2012), version 4.10.2^2^. We employed the planimetric contouring method (Rombaux et al., 2009). The examiner manually delineates the OBs in the coronal slices and multiplies each surface area (mm^2^) by slice thickness (1 mm). The posterior end of the OBs is reached when encountering two equal sized, narrower surface areas in a row. Finally, all obtained volumes are added up for the total volume of each bulb. OB measurements were performed by three independent experimenters who were blinded for the weight status and sex of the participants. The mean of their results serves as the OB volume.

First, we applied a group design to display differences between BMI groups as defined by international standards on OB volume while controlling for sex. Assumption tests showed homogeneity of variances/covariances, as assessed by Box’s M test. There were no univariate/multivariate outliers, as assessed by standardized residuals greater than ±3 standard deviations/Mahalanobis distance. Second, the relationship between OB volume and measures that reflect body weight status (BMI, WHR, body fat percentage, leptin) was assessed by correlation analysis. We performed partial Spearman’s rank correlation for not normally distributed variables (BMI, WHR, leptin, insulin, HOMA-IR, age, TDI score, odor identification, odor discrimination, craving questionnaires, eating behavior questionnaires, olfaction questionnaires) with OB volume. Since OB volume is smaller in women when compared to men, we used sex as a covariate. Additionally, we used partial Pearson’s product moment correlation to depict the relation between normally distributed variables (body fat percentage, odor threshold, DFS data). We interpreted the strength of the correlation according to Cohen’s conventions (Cohen, 1988).

We further used SPSS (version 22.0, SPSS Inc., Chicago, IL) for the replication of a mediation analysis to disentangle the effect of insulin resistance on the relationship between obesity and olfactory function. Unstandardized indirect effects were computed for each of 10,000 bootstrapped samples, and the 95% confidence interval was computed by determining the indirect effects at the 2.5th and 97.5th percentiles.

## Results

### Sample Characteristics

Participant characteristics are given in Table 1. Descriptive values for the total sample as well as for BMI subgroups are listed. Table 1 contains general characteristics (age, depressive symptoms, information about passive smoking), metabolic health parameters (BMI, WHR, body fat percentage) and hormonal parameters (plasma insulin, glucose and leptin, HOMA-IR score). BDI scores indicated no or only mild depressive symptoms in all subjects (mean = 3.01, *SD* = 3.35, range: 0–13), however, participants with obesity had significantly more depressive symptoms than participants with normal weight. As expected participants significantly differed in BMI, WHR, and body fat percentage. Participants with normal weight had also significantly lower HOMA-IR scores as well as lower levels of plasma insulin, glucose and leptin when compared to obese participants.

### Psychophysical Function

Olfactory function assessed by Sniffin’ Sticks and food associated odor test (FAOT) are given in Table 2. A one-way multivariate analysis of variance was run to determine the effect of weight status (normal weight vs. obese) on olfactory performance (Odor identification, odor discrimination, odor identification, TDI sum score) while controlling for sex. There was no statistically significant difference between weight groups in all olfactory tests, *F*(3, 51) = 0.389, *p* = 0.761 (Table 2).

**TABLE 2:** Olfactory performance assessed with the Sniffin’ Sticks battery and FAOT.

	Total (*n* = 67)	Correlation with BMI	Correlation with OB volume	Normal weight (*n* = 28)	Obese (*n* = 28)	*p*-value/*F*-value between NW and OB	Overweight (*n* = 11)

	Mean ± SD (range)	*p*-value/r		Mean ± SD (range)			
**Smell tests**							
Threshold	7.63 ± 1.85 (1.75 − 11.00)	0.393/ − 0.109^*a*^	0.348/0.120^*a*^	7.65 ± 2.19 (1.75 − 11.00)	7.56 ± 1.61 (4.25 − 10.50)	0.959/0.030^*b*^	7.75 ± 1.65
Discrimination	12.45 ± 2.12 (6 − 16)	0.938/0.010^*a*^	0.505/0.086^*a*^	12.11 ± 2.67 (6 − 16)	12.64 ± 1.66 (10 − 16)	0.863/0.812^b^	12.82 ± 1.54
Identification	13.55 ± 1.51 (9 − 16)	0.268/-0.142^*a*^	0.807/0.003^*a*^	13.54 ± 1.17 (11 − 15)	13.43 ± 1.91 (9 − 16)	0.801/0.064^b^	13.91 ± 1.14
TDI sumscore	33.63 ± 3.90 (22.75 − 40.25)	0.230/-0.133^*a*^	0.244/0.149^*a*^	33.29 ± 4.60 (22.75 − 40.25)	33.63 ± 3.54 (26.25 − 38.50)	0.758/0.096^b^	34.48 ± 2.85
FAOT	13.4 ± 1.47 (9 − 16)	0.272/-0.141^*a*^	0.854/0.024^*a*^	13.29 ± 1.27 (10 − 15)	13.39 ± 1.50 (11 − 16)	0.774/0.083^b^	13.73 ± 1.90

**Data are presented as mean values, standard deviations, and minimum and maximum values. ^*a*^Spearman’s rank-order correlation. ^*b*^One-way analysis of variance with the covariate sex (ANCOVA). BMI, body mass index; TDI sumscore, sumscore of odor threshold, odor discrimination, and odor identification; FAOT, food associated odor test.**

In addition, none of the olfactory tests was associated with OB volume or BMI as determined by Pearson’s product moment correlation and Spearman’s rank-order correlation (Table 2).

### Relationship Between OB Volume and BMI/Other Measures That Are Associated With Metabolic Health (WHR, WHtR, Body Fat Percentage, FMI, Leptin, Insulin Resistance)

**FIGURE 1:**
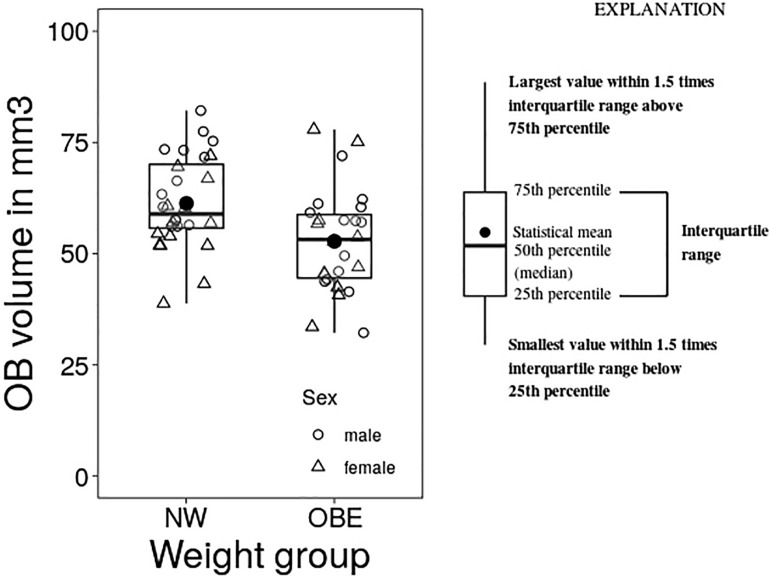
Group differences between normal weight (NW) and obese (OBE) participants in OB volume as assessed by one-way analysis of variance with the covariate sex (ANCOVA).

Means, range and standard deviations of OB volume and whole brain volume grouped by weight status and in total are depicted in Table 3. Firstly, we explored group differences in OB volume between participants with obesity and normal weight, we found a significant group effect as determined by one-way ANCOVA [*F*(1, 52) = 8.119, *p* = 0.004] with smaller volume in individuals with obesity (Figure 1). Additionally, we checked whole brain volume to control the specificity of this effect. There was no difference in whole brain volume in the weight groups [*F*(1, 52) = 1,691, *p* = 0.682].

**TABLE 3:** Olfactory bulb (OB) and whole brain volume (WBV) overall participants and for weight groups.

	Total	Normal weight (*n* = 28)	Obese (*n* = 26)	*p*-value/*F*-value between NW and OBE	Overweight

	Mean ± SD (range)				
OB volume right	56.95 ± 12.83 (26.21 − 82.47)	61.01 ± 10.67 (26.21 − 82.47)	52.05 ± 10.94 (30.59 − 74.87)	0.002**/9.272^*a*^	58.19 ± 18.33
OB volume left	57.59 ± 13.35 (22.59 − 84.25)	61.58 ± 11.25 (22.59 − 82.74)	53.51 ± 13.07 (32.44 − 80.90)	0.016*/5.959^*a*^	57.07 ± 16.83
Mean OB volume	57.27 ± 12.64 (24.40 − 82.13)	61.29 ± 10.33 (24.40 − 80.83)	52.78 ± 11.64 (32.30 − 77.89)	0.004**/8.119^*a*^	57.63 ± 17.27
WBV	1.20 ± 0.10 (0.96 − 1.39)	1.21 ± 0.10 (1.04 − 1.33)	1.20 ± 0.10 (1.00 − 1.39)	0.682/.169^*a*^	1.18 ± 0.08

*^*a*^One-way analysis of variance with the covariate sex (ANCOVA). OB volume in mm^3^, WBV in liter; NW, normal weight; OBE, obese. ^∗^*p*≤0.05; ^∗∗^*p*≤0.01; Bold font indicates statistical significance.*

**FIGURE 2:**
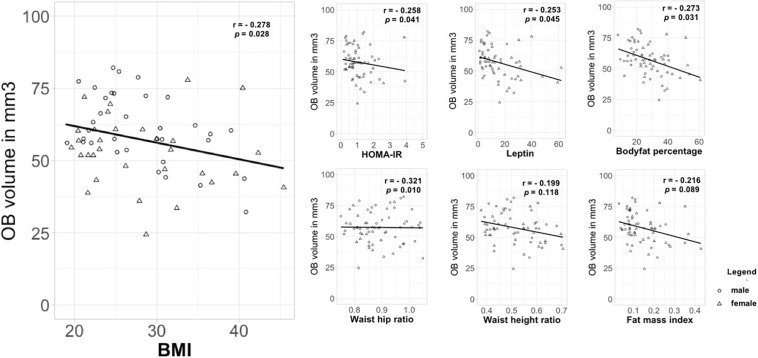
Correlation between OB volume and measures that are associated with metabolic health status in obesity. OB volume in mm^3^; BMI in kg/m^2^; HOMA-IR, homeostatic model assessment of insulin resistance; leptin in ng/ml; body fat in percentage; WHtR, waist-height ratio; WHR, waist-hip ratio in cm; FMI, fat mass index.

Additionally, we investigated the relationship between OB volume and BMI as a continuous variable as well as other measures of metabolic health in obesity using correlation analysis. A partial Spearman’s rank-order correlation was run to determine the relationship between BMI and OB volume whilst controlling for sex. There was a weak, negative correlation between OB volume and BMI, which was statistically significant (*r* = -0.278, *n* = 65, *p* = 0.028) (Figure 2). Further, we assessed other measures that are associated with metabolic health status in obesity: HOMA-IR, leptin, body fat percentage, WHtR, WHR, and FMI (Figure 2). A partial Pearson’s product moment correlation was run to determine the relationship between body fat percentage and OB volume. There was a weak, negative correlation between OB volume and body fat percentage, which was statistically significant (*r* = -0.273, *n* = 65, *p* = 0.031). A partial Spearman’s rank-order correlation was run to determine the relationship between OB volume and HOMA-IR/leptin/WHtR/WHR/FMI whilst controlling for sex. There were statistically significant weak to moderate, negative correlations for HOMA-IR and OB volume (*r* = -0.258, *n* = 65, *p* = 0.041), leptin and OB volume (*r* = -0.253, *n* = 65, *p* = 0.045) as well as for WHtR and OB volume (*r* = -0.321, *n* = 65, *p* = 0.010). However, there were no statistically significant correlations between OB volume and WHR (*r* = -0.199, *n* = 65, *p* = 0.118) or FMI (*r* = -0.216, *n* = 65, *p* = 0.089) whilst controlling for sex.

### Replication: Mediation Effect of HOMA-IR on the Relation Between BMI and Smell Function

The relationship between BMI and olfactory function, as assessed by TDI score, was mediated by HOMA-IR. The standardized regression coefficient was significant between BMI and the mediator HOMA-IR (*r* = 0.625, *p* = 0.001) and between TDI score and the mediator HOMA-IR (*r* = 0.348, *p* = 0.042). The standardized indirect effect between BMI and TDI score via HOMA-IR was -0.154 (CI -0.268 and -0.036). There was no significant direct effect for BMI and TDI score (*r* = 0.126, *p* = 0.164, CI: -0.053 and 0.304).

### Relation Between Eating Behavior and BMI/OB Volume

A Spearman’ rank-order correlation was applied to investigate the relationship between BMI and eating behavior questionnaires. We found a weak, positive correlation between BMI and cognitive restraint as well as hunger scale of the TFEQ (*r* = 0.258, *n* = 61, *p* = 0.041; *r* = 0.249, *n* = 61, *p* = 0.049).

A partial Spearman’s rank-order correlation was run to determine the relationship between eating behavior questionnaire data and OB volume whilst controlling for sex. There was a weak, positive correlation between OB volume and chocolate craving (sum score from the FCQ-Trait Chocolate questionnaire), which was statistically significant (*r* = 0.278, *n* = 59, *p* = 0.033). All correlational data of BMI and OB volume with eating behavior questionnaires are given in Supplementary Table 1.

Yet we treat these findings with caution, since they would not survive alpha level correction for multiple testing.

## Discussion

In this study, we demonstrate for the first time that OB volume is reduced in individuals with obesity when compared to those with normal weight. Additionally, we found weak to moderate negative correlations between OB volume and BMI as well as other measures of metabolic health in obesity, such as body fat percentage, WHtR, leptin levels and insulin resistance. Our results imply that, similar to other diseases such as depression and Parkinson’s disease (Negoias et al., 2010; Li et al., 2016), obesity also involves a neuroanatomical change in the OBs compared to healthy participants with normal weight.

Since we could show that whole brain volume was not associated with BMI and weight status in our sample, we conclude that our observation is not related to a general atrophy in our obese sample but might be specific to the olfactory system. However, since we have not measured the volume of other sensory regions, such as the gustatory, visual or auditory cortex, we cannot conclude with certainty that our results are specific to this one sensory system or might also affect other sensory areas. Additionally, as our study groups did not differ in age, we can exclude a frequently observed effect of age on the sense of smell in obesity due to older obese study populations when compared to those of normal weight, as discussed in a recent review paper by Peng and colleagues (Peng et al., 2019).

Yet, what might be the underlying causes for smaller OB volume in obesity? Our observation poses the question whether OB volume reduction is involved in the development of obesity or a consequence of this condition. First, it might be possible that altered olfactory processing leads to changes in eating behavior and consequently favors weight gain. Second, it might be possible that reduction in OB volume is a consequence of obesity and is for instance caused by metabolic and endocrine impairments. Both questions cannot be fully addressed by the design of our study. Nonetheless, we assume that the latter point of view is more likely, since it has been shown that olfactory dysfunction is more pronounced in severe obesity when compared to overweight or moderate obesity (Richardson et al., 2004; Pastor et al., 2016; Fernandez-Garcia et al., 2017).

Surprisingly, we could not find a relationship between OB volume or BMI and psychophysiological measures of olfactory ability, respectively. This is striking for two reasons: (1) obesity is commonly associated with low olfactory ability and (2) we expected that lower OB volume in obesity is associated with lower olfactory function. As to the first point, we think that our very young and healthy obese sample may be the reason for the preserved olfactory ability. As discussed by the authors of this paper it is plausible that individuals with obesity that are metabolically healthy may have normal olfactory function (Poessel et al., 2020). This point of view is in line with another finding that especially people that are morbidly obese when compared to moderately obese are affected from limitations in olfactory function (Richardson et al., 2004). In this regard, proposed explanatory models of impaired olfactory function in obesity might explain this phenomenon: They point to an important role of metabolic and hormonal changes in obesity that may cause altered smell perception (Fernandez-Garcia et al., 2017; Peng et al., 2019). For instance, leptin, which is often increased in obesity while their brain is insensitive to this hormone, has an inhibitory role on olfactory function in mice (Getchell et al., 2006). In line with this reasoning, it is interesting that we could replicate our results from Poessel et al. (2020) in this sample. We find a strong mediating effect of insulin resistance as assessed by HOMA-IR score on the relationship between BMI and olfactory function as assessed by TDI score. This implies that high BMI is associated with lower olfactory function via IR. As to the second point, one can speculate that reduced OB volume in obesity is an early sign of the pathophysiological changes in olfactory function. Therefore, obesity and associated metabolic changes might have negative effects on the olfactory system before hampering obvious psychophysical function. Especially, young age, short duration of obesity and being metabolically healthy might protect against decline of olfactory function (Attems et al., 2015; Lacroix et al., 2015; Riera and Dillin, 2016). Yet, it is unexpected that the alteration of smell perception does not first manifest itself in behavior, but in the brain anatomy. Typically, a bottom-up mechanism is discussed as the cause of decreasing OB volume, for instance in chronic rhinosinusitis (Han et al., 2017) and smoking (Schriever et al., 2013). In those conditions, lower afferent input is thought to reduce OB volume because of inflammatory processes in the nose or due to toxic effect of smoke-associated products on the olfactory epithelium. This bottom-up mechanism of olfactory dysfunction has been shown in animal models: deprivation of olfactory function leads to decreased OB volume (Cummings et al., 1997). Interestingly, this effect is reversible after restoring olfactory function. In line with that result is an observation in humans, where surgical treatment of patients with chronic rhinosinusitis results in increasing OB volume (Gudziol et al., 2009). However, since we detect a low OB volume without obvious olfactory dysfunction in our obese sample, it might be possible that function is preserved due to redundant information processing: the specific anatomy and physiology of the olfactory system with its parallel processing pathways could ensure that olfactory function is maintained. Olfactory sensory neurons project to homologous glomeruli on the level of the olfactory bulb, in a way that odor information is represented and processed in two mirror maps (Nagao et al., 2002; Sato et al., 2020). From there, the olfactory information is transferred to higher order olfactory brain regions in a parallel manner (Savic et al., 2000; Nagayama et al., 2010; Payton et al., 2012). With regard to our data, we assume that based on the parallel circuitry of odor information processing at the level of the olfactory bulb, a reduction in the volume of OBs does not necessarily lead to behavioral deficits.

Secondly, a top-down process explanation seems also probable, as for instance discussed in Parkinson’s Disease (Li et al., 2016) and depression (Negoias et al., 2010). Here, neurodegenerative processes or disrupted salience processing in the brain with a bias toward internal thoughts disregarding the exteroceptive sensory system may lead to a reduction of OB volume (Rottstädt et al., 2018). Especially, the loss of sensory neurons in obesity (O’Brien et al., 2017) might result in a decrease of sensory function. This has been shown in various sensory systems, such as the auditory (Hwang et al., 2013) and olfactory system. More specifically, Thiebaud et al. (2014) reported that hyperlipidemic diet in mice and associated obesity leads to a loss of olfactory sensory neurons and a decrease in olfactory function (Thiebaud et al., 2014). Moreover, it might be possible that the neural response to sensory input is blunted as it has been shown in taste processing (Weiss et al., 2019).

It has recently been shown that hormones whose homeostatic signaling may be impaired in obesity (Baly et al., 2007; Aime et al., 2012; Russo et al., 2018), modulate olfactory performance in humans. More specifically, intranasally applied insulin as well as intravenously applied ghrelin improve olfactory function (Tong et al., 2011; Thanarajah et al., 2019). Interestingly, the OBs have a high density of receptors for these hormones. Thus, the OBs might be directly affected by these alterations. In this respect, we think that another approach to explain diminished OB volume in the absence of olfactory dysfunction might be plausible: metabolic and hormonal dysfunction in obesity might directly affect the neurogenesis and synaptogenesis of the OB. Interestingly, chronic inflammation, as observed in obesity, is associated with the disruption of hippocampal neurogenesis (Chesnokova et al., 2016) and lower hippocampal and gray matter volumes (Tsai et al., 2019). Especially, body fat percentage, leptin and insulin resistance are among other indicators of increased fat mass and might thus be associated with chronic low-grade inflammation in obesity (Jung and Choi, 2014; Chen et al., 2015; Saad et al., 2016; La Cava, 2017; Reilly and Saltiel, 2017). Interestingly, it has been found in rats that diet induced type 2 diabetes leads to a decreased electrophysiological response of olfactory neurons (Rivière et al., 2016). Moreover, obesity and chronically high levels of insulin disrupt the metabolic sensing of the OBs in mice (Fadool et al., 2011). In the light of these findings, one can speculate that the negative correlation between OB volume and markers of metabolic health might be a hint that neurogenesis of the OBs might be affected by obesity associated inflammatory processes.

Another, albeit highly speculative, possibility could be that smells might be differently processed in individuals with obesity. Following the observation of Weiss et al. (2020) that people without apparent OBs can still smell, one can speculate that other structures in the brain might take over the processing of olfactory information by the formation of a glomerular space somewhere else in the cortex. In this respect, we assume that although the OB volume might be lower in our obese sample, the olfactory ability might be preserved by other structures.

Since the OBs are structures that respond directly to odors and play an important role in the processing and dissemination of olfactory information to higher order brain regions (Rombaux et al., 2009), they may also play a crucial role in homeostatic signaling and eating behavior. Thus, we examined the relationship between OB volume and eating behavior as assessed by subjective questionnaires on cravings (FCQ), actual food intake (DFS) and eating style (TFEQ). We found weak positive correlations between OB volume and chocolate craving whilst controlling for sex and BMI. This means that the higher chocolate craving the bigger are the OBs. These results should be interpreted prudently since this correlation would not survive alpha level correction for multiple testing. However, we think this is a first interesting link between brain anatomy in the olfactory system and eating behavior.

Although we have planned our study carefully, there are limitations to consider. First, in the context of eating behavior and obesity we would encourage researchers to develop or use further instruments to assess olfactory perception. Albeit the standard olfactory Sniffin’ Sticks test battery is a well-validated measure of olfactory function, it might not be as sensitive to depict subtle differences in healthy subjects, because it was primarily developed for the evaluation of olfactory loss. Moreover, the investigation of eating behavior can be extended in future studies. Especially, we would suggest that direct and implicit measurements of eating behavior should be carried out to avoid bias through subjective reporting. In addition, we consider it necessary to determine the onset and duration of obesity in future studies in order to make a more reliable statement about the influence of obesity associated changes in the metabolic and endocrine systems on olfactory function. In this context, we think an extension of the hormonal profile and collecting inflammatory markers would be compelling. Additionally, we think that our results only provide first indications that the olfactory system is neuroanatomically altered in people with obesity. Further studies should also look at other structures of the primary and secondary olfactory pathways to understand whether reduction in OB volume also affects higher regions of olfactory processing.

In sum, our study finds solid evidence that OB volume is decreased in obesity while sensory function appears to be preserved. Furthermore, we show negative correlations between OB volume and insulin resistance, leptin levels and body fat. This might provide a mechanistic link between changes in the olfactory system in obesity.

## Data Availability Statement

The raw data supporting the conclusions of this article will be made available by the authors, without undue reservation.

## Ethics Statement

The studies involving human participants were reviewed and approved by the Ethics Committee of the University of Leipzig (Reference number 387/17-ek, date of vote: 2017–10–17). The patients/participants provided their written informed consent to participate in this study.

## Author Contributions

MP, TH, and AH: conceptualization. MP, NB, and AH: methodology. MP and NB: software, investigation, and writing—original draft preparation. MP: validation, data curation, and visualization. MP, AJ, and NB: formal analysis. AH and AV: resources. MP, AH, TH, NB, and AV: writing—review and editing. AH, TH, and AV: supervision. MP and AH: project administration and funding acquisition. All authors have read and agreed to the published version of the manuscript.

## Conflict of Interest

The authors declare that the research was conducted in the absence of any commercial or financial relationships that could be construed as a potential conflict of interest.

## Footnotes

1 https://www.dtu.ox.ac.uk/homacalculator/index.php

2 https://www.slicer.org/

## Supplementary Material

Supplementary Table 1 Correlation analysis of eating behavior questionnaires and BMI/OB volume.

## References

[B1] Aime P. Hegoburu C. Jaillard T. Degletagne C. Garcia S. Messaoudi B. (2012). A physiological increase of insulin in the olfactory bulb decreases detection of a learned aversive odor and abolishes food odor-induced sniffing behavior in rats. *PLoS One* 7:e51227. 10.1371/journal.pone.0051227 23251461 PMC3522659

[B2] Attems J. Walker L. Jellinger K. A. (2015). Olfaction and aging: a mini-review. *Gerontology* 61 485–490. 10.1159/000381619 25968962

[B3] Baly C. Aioun J. Badonnel K. Lacroix M.-C. Durieux D. Schlegel C. (2007). Leptin and its receptors are present in the rat olfactory mucosa and modulated by the nutritional status. *Brain Res.* 1129 130–141. 10.1016/j.brainres.2006.10.030 17169337

[B4] Baskin D. G. Porte D. Guest K. Dorsa D. M. (1983). Regional concentrations of insulin in the rat brain. *Endocrinology* 112 898–903. 10.1210/endo-112-3-898 6337049

[B5] Beck A. T. Ward C. H. Mendelson M. Mock J. Erbaugh J. (1961). An inventory for measuring depression. *Arch Gen Psychiatry* 4 561–571. 10.1001/archpsyc.1961.01710120031004 13688369

[B6] Berthoud H. R. (2012). The neurobiology of food intake in an obesogenic environment. *Proc. Nutr. Soc.* 71 478–487. 10.1017/S0029665112000602 22800810 PMC3617987

[B7] Blüher M. (2020). Metabolically healthy obesity. *Endocr. Rev.* 41 405–420. 10.1210/endrev/bnaa004 32128581 PMC7098708

[B8] Brunner Y. F. Benedict C. Freiherr J. (2013). Intranasal insulin reduces olfactory sensitivity in normosmic humans. *J. Clin. Endocrinol. Metab.* 98 E1626–E1630. 10.1210/jc.2013-2061 23928664

[B9] Buschhüter D. Smitka M. Puschmann S. Gerber J. C. Witt M. Abolmaali N. D. (2008). Correlation between olfactory bulb volume and olfactory function. *Neuroimage* 42 498–502. 10.1016/j.neuroimage.2008.05.004 18555701

[B10] Chen L. Chen R. Wang H. Liang F. (2015). Mechanisms linking inflammation to insulin resistance. *Int. J. Endocrinol.* 2015 1–9. 10.1155/2015/508409 26136779 PMC4468292

[B11] Chesnokova V. Pechnick R. N. Wawrowsky K. (2016). Chronic peripheral inflammation, hippocampal neurogenesis, and behavior. *Brain Behav. Immun.* 58 1–8. 10.1016/j.bbi.2016.01.017 26802985 PMC4956598

[B12] Christen T. Trompet S. Noordam R. van Klinken J. B. van Dijk K. W. Lamb H. J. (2018). Sex differences in body fat distribution are related to sex differences in serum leptin and adiponectin. *Peptides* 107 25–31. 10.1016/j.peptides.2018.07.008 30076861

[B13] Cohen J. (1988). *Statistical Power Analysis for the Behavioral Sciences*, 2 Edn Hillsdale, NJ: L. Erlbaum Associates, 10.4324/9780203771587

[B14] Cummings D. M. Henning H. E. Brunjes P. C. (1997). Olfactory bulb recovery after early sensory deprivation. *J. Neurosci.* 17 7433–7440. 10.1523/jneurosci.17-19-07433.1997 9295389 PMC6573448

[B15] Denzer-Lippmann M. Y. Beauchamp J. Freiherr J. Thuerauf N. Kornhuber J. Buettner A. (2017). Development and validation of a food-associated olfactory test (FAOT). *Chem. Senses* 42 47–57. 10.1093/chemse/bjw099 27681497

[B16] Derntl B. Schopf V. Kollndorfer K. Lanzenberger R. (2013). Menstrual cycle phase and duration of oral contraception intake affect olfactory perception. *Chem. Senses* 38 67–75. 10.1093/chemse/bjs084 23071141 PMC3522517

[B17] Durham A. E. (2016). Insulin dysregulation and obesity: you are what you eat. *Vet. J.* 213:90. 10.1016/j.tvjl.2016.03.010 27240923

[B18] Fadool D. A. Tucker K. Pedarzani P. (2011). Mitral cells of the olfactory bulb perform metabolic sensing and are disrupted by obesity at the level of the Kv1.3 ion channel. *PLoS One* 6:e24921. 10.1371/journal.pone.0024921 21966386 PMC3178571

[B19] Fedorov A. Beichel R. Kalpathy-Cramer J. Finet J. Fillion-Robin J.-C. Pujol S. (2012). 3D Slicer as an image computing platform for the Quantitative Imaging Network. *Magn. Reson. Imaging* 30 1323–1341. 10.1016/j.mri.2012.05.001 22770690 PMC3466397

[B20] Fernández-Aranda F. Agüera Z. Fernández-García J. C. Garrido-Sanchez L. Alcaide-Torres J. Tinahones F. J. (2016). Smell-taste dysfunctions in extreme weight/eating conditions: Analysis of hormonal and psychological interactions. *Endocrine* 51 256–267. 10.1007/s12020-015-0684-9 26198367

[B21] Fernandez-Garcia J. C. Alcaide J. Santiago-Fernandez C. Roca-Rodriguez M. M. Aguera Z. Baños R. (2017). An increase in visceral fat is associated with a decrease in the taste and olfactory capacity. *PLoS One* 12:e0171204. 10.1371/journal.pone.0171204 28158237 PMC5291407

[B22] Firmin M. W. Gillette A. L. Hobbs T. E. Wu D. (2016). Effects of olfactory sense on chocolate craving. *Appetite* 105 700–704. 10.1016/j.appet.2016.07.004 27395410

[B23] Fromm S. P. Horstmann A. (2019). Psychometric evaluation of the German version of the dietary fat and free sugar–short questionnaire. *Obes. Facts* 12, 518–528. 10.1159/000501969 31553993 PMC6876588

[B24] Gaillet-Torrent M. Sulmont-Rosse C. Issanchou S. Chabanet C. Chambaron S. (2014). Impact of a non-attentively perceived odour on subsequent food choices. *Appetite* 76 17–22. 10.1016/j.appet.2014.01.009 24462492

[B25] Getchell T. V. Kwong K. Saunders C. P. Stromberg A. J. Getchell M. L. (2006). Leptin regulates olfactory-mediated behavior in ob/ob mice. *Physiol. Behav.* 87 848–856. 10.1016/j.physbeh.2005.11.016 16549076

[B26] Gudziol V. Buschhuter D. Abolmaali N. Gerber J. Rombaux P. Hummel T. (2009). Increasing olfactory bulb volume due to treatment of chronic rhinosinusitis–A longitudinal study. *Brain* 132 3096–3101. 10.1093/brain/awp243 19773353

[B27] Gutch M. Kumar S. Razi S. M. Gupta K. K. Gupta A. (2015). Assessment of insulin sensitivity/resistance. *Indian J. Endocrinol. Metab.* 19 160–164. 10.4103/2230-8210.146874 25593845 PMC4287763

[B28] Han P. Whitcroft K. L. Fischer J. Gerber J. Cuevas M. Andrews P. (2017). Olfactory brain gray matter volume reduction in patients with chronic rhinosinusitis: gray matter loss in CRS. *Int. Forum Allergy Rhinol.* 7 551–556. 10.1002/alr.21922 28383208

[B29] Havrankova J. Brownstein M. Roth J. (1981). Insulin and insulin receptors in rodent brain. *Diabetologia* 20 268–273. 10.1007/BF00254492 7014325

[B30] Hedner M. Larsson M. Arnold N. Zucco G. M. Hummel T. (2010). Cognitive factors in odor detection, odor discrimination, and odor identification tasks. *J. Clin. Exp. Neuropsychol.* 32 1062–1067. 10.1080/13803391003683070 20437286

[B31] Hoaglin D. C. Iglewicz B. (1987). Fine-tuning some resistant rules for outlier labeling. *J. Am. Stat. Assoc.* 82 1147–1149. 10.1080/01621459.1987.10478551

[B32] Hummel T. Kobal G. Gudziol H. Mackay-Sim A. (2007). Normative data for the “Sniffin’ Sticks” including tests of odor identification, odor discrimination, and olfactory thresholds: An upgrade based on a group of more than 3,000 subjects. *Eur. Arch. Otorhinolaryngol.* 264 237–243. 10.1007/s00405-006-0173-0 17021776

[B33] Hummel T. Sekinger B. Wolf S. R. Pauli E. Kobal G. (1997). ,,Sniffin“ sticks’: Olfactory performance assessed by the combined testing of odor identification, odor discrimination and olfactory threshold. *Chem. Senses* 22 39–52. 9056084 10.1093/chemse/22.1.39

[B34] Hwang J.-H. Hsu C.-J. Yu W.-H. Liu T.-C. Yang W.-S. (2013). Diet-induced obesity exacerbates auditory degeneration via hypoxia, inflammation, and apoptosis signaling pathways in CD/1 Mice. *PLoS One* 8:e60730. 10.1371/journal.pone.0060730 23637762 PMC3637206

[B35] Jung U. J. Choi M. S. (2014). Obesity and its metabolic complications: The role of adipokines and the relationship between obesity, inflammation, insulin resistance, dyslipidemia and nonalcoholic fatty liver disease. *Int. J. Mol. Sci.* 15 6184–6223. 10.3390/ijms15046184 24733068 PMC4013623

[B36] Kitamura A. Torii K. Uneyama H. Niijima A. (2010). Role played by afferent signals from olfactory, gustatory and gastrointestinal sensors in regulation of autonomic nerve activity. *Biol. Pharm. Bull.* 33 1778–1782. 10.1248/bpb.33.1778 21048298

[B37] Kobal G. Hummel T. Sekinger B. Barz S. Roscher S. Wolf S. (1996). “Sniffin’ sticks”: screening of olfactory performance. *Rhinology* 34 222–226. 9050101

[B38] La Cava A. (2017). Leptin in inflammation and autoimmunity. *Cytokine* 98 51–58. 10.1016/j.cyto.2016.10.011 27916613 PMC5453851

[B39] Lacroix M.-C. Caillol M. Durieux D. Monnerie R. Grebert D. Pellerin L. (2015). Long-lasting metabolic imbalance related to obesity alters olfactory tissue homeostasis and impairs olfactory-driven behaviors. *Chem. Senses* 40 537–556. 10.1093/chemse/bjv039 26209545

[B40] Landecho M. F. Tuero C. Valentí V. Bilbao I. de la Higuera M. Frühbeck G. (2019). Relevance of leptin and other adipokines in obesity-associated cardiovascular risk. *Nutrients* 11:2664. 10.3390/nu11112664 31694146 PMC6893824

[B41] Lean M. E. J. Malkova D. (2016). Altered gut and adipose tissue hormones in overweight and obese individuals: cause or consequence? *Int. J. Obes.* 40 622–632. 10.1038/ijo.2015.220 26499438 PMC4827002

[B42] Li J. Gu C. Su J. Zhu L. Zhou Y. Huang H. (2016). Changes in olfactory bulb volume in parkinson’s disease: a systematic review and meta-analysis. *PLoS One* 11:e0149286. 10.1371/journal.pone.0149286 26900958 PMC4762676

[B43] Liu H. Hang W. Liu G. Han T. (2017). [Olfactory bulb volume in patients with posttraumatic olfactory dysfunction]. *Zhonghua Er Bi Yan Hou Tou Jing Wai Ke Za Zhi* 52 273–277. 10.3760/cma.j.issn.1673-0860.2017.04.007 28441804

[B44] Liu P. Ma F. Lou H. Liu Y. (2013). The utility of fat mass index vs. Body mass index and percentage of body fat in the screening of metabolic syndrome. *BMC Public Health* 13:629. 10.1186/1471-2458-13-629 23819808 PMC3703297

[B45] Loeffler M. Engel C. Ahnert P. Alfermann D. Arelin K. Baber R. (2015). The LIFE-Adult-Study: Objectives and design of a population-based cohort study with 10,000 deeply phenotyped adults in Germany. *BMC Public Health* 15:691. 10.1186/s12889-015-1983-z 26197779 PMC4509697

[B46] Łopatyński J. Mardarowicz G. Szcześniak G. (2003). A comparative evaluation of waist circumference, waist-to-hip ratio, waist-to-height ratio and body mass index as indicators of impaired glucose tolerance and as risk factors for type-2 diabetes mellitus. *Ann. Univ. Mariae Curie Sklodowska Med.* 58 413–419. 15315025

[B47] Lopez-Gonzalez D. Partida-Gaytán A. Wells J. C. Reyes-Delpech P. Avila-Rosano F. Ortiz-Obregon M. (2020). Obesogenic lifestyle and its influence on adiposity in children and adolescents, evidence from Mexico. *Nutrients* 12:819. 10.3390/nu12030819 32204522 PMC7146202

[B48] Marks J. L. Porte D. Stahl W. L. Baskin D. G. (1990). Localization of insulin receptor mRNA in rat brain by in situ hybridization. *Endocrinology* 127 3234–3236. 10.1210/endo-127-6-3234 2249648

[B49] Matthews D. R. Hosker J. P. Rudenski A. S. Naylor B. A. Treacher D. F. Turner R. C. (1985). Homeostasis model assessment: insulin resistance and β-cell function from fasting plasma glucose and insulin concentrations in man. *Diabetologia* 28 412–419. 10.1007/BF00280883 3899825

[B50] Mazal P. P. Haehner A. Hummel T. (2016). Relation of the volume of the olfactory bulb to psychophysical measures of olfactory function. *Eur. Arch. Oto Rhino Laryngol.* 273 1–7. 10.1007/s00405-014-3325-7 25308243

[B51] McNeil J. Cameron J. D. Finlayson G. Blundell J. E. Doucet E. (2013). Greater overall olfactory performance, explicit wanting for high fat foods and lipid intake during the mid-luteal phase of the menstrual cycle. *Physiol. Behav.* 112–113 84–89. 10.1016/j.physbeh.2013.02.008 23458628

[B52] Meule A. Teran C. B. Berker J. Gründel T. Mayerhofer M. Platte P. (2014). On the differentiation between trait and state food craving: Half-year retest-reliability of the *Food Cravings Questionnaire-Trait-reduced* (FCQ-T-r) and the *Food Cravings Questionnaire-State* (FCQ-S). *J. Eat. Disord.* 2. 10.1186/s40337-014-0025-z 25356313 PMC4212121

[B53] Meule A. Hormes J. M. (2015). Chocolate versions of the *Food Cravings Questionnaires*. Associations with chocolate exposure-induced salivary flow and ad libitum chocolate consumption. *Appetite* 91 256–265. 10.1016/j.appet.2015.04.054 25913686

[B54] Mueller C. Renner B. (2006). A new procedure for the short screening of olfactory function using five items from the ,,Sniffin’ Sticks“ identification test kit. *Am. J. Rhinol.* 20 113–116. 16539306

[B55] Mugler J. P. Brookeman J. R. (1990). Three-dimensional magnetization-prepared rapid gradient-echo imaging (3D MP RAGE). *Magn. Reson. Med.* 15 152–157. 10.1002/mrm.1910150117 2374495

[B56] Münzberg H. Heymsfield S. B. (2015). “Leptin, obesity, and leptin resistance,” in *Leptin—Regulation and Clinical Applications*, ed. Sam Dagogo-Jack M. D. (Cham: Springer International Publishing), 67–78. 10.1007/978-3-319-09915-6_6

[B57] Murphy K. G. Bloom S. R. (2006). Gut hormones and the regulation of energy homeostasis. *Nature* 444 854–859. 10.1038/nature05484 17167473

[B58] Nagl M. Hilbert A. de Zwaan M. Braehler E. Kersting A. (2016). The German version of the dutch eating behavior questionnaire: psychometric properties, measurement invariance, and population-based norms. *PLOS ONE* 11:e0162510. 10.1371/journal.pone.0162510 27656879 PMC5033316

[B59] Nagao H. Yamaguchi M. Takahash Y. Mori K. (2002). Grouping and representation of odorant receptors in domains of the olfactory bulb sensory map. *Microsc. Res. Tech.* 58:168. 10.1002/jemt.10146 12203695

[B60] Nagayama S. Enerva A. Fletcher M. L. Masurkar A. V. Igarashi K. M. Mori K. (2010). Differential axonal projection of mitral and tufted cells in the mouse main olfactory system. *Front. Neural Circuits* 4:120. 10.3389/fncir.2010.00120 20941380 PMC2952457

[B61] Negoias S. Croy I. Gerber J. Puschmann S. Petrowski K. Joraschky P. (2010). Reduced olfactory bulb volume and olfactory sensitivity in patients with acute major depression. *Neuroscience* 169 415–421. 10.1016/j.neuroscience.2010.05.012 20472036

[B62] O’Brien P. D. Hinder L. M. Callaghan B. C. Feldman E. L. (2017). Neurological consequences of obesity. *Lancet Neurol.* 16 465–477. 10.1016/S1474-4422(17)30084-4 28504110 PMC5657398

[B63] Pastor A. Fernandez-Aranda F. Fito M. Jimenez-Murcia S. Botella C. Fernandez-Real J. M. (2016). A lower olfactory capacity is related to higher circulating concentrations of Endocannabinoid 2-arachidonoylglycerol and higher body mass index in women. *PLoS One* 11:e0148734. 10.1371/journal.pone.0148734 26849214 PMC4746072

[B64] Payton C. A. Wilson D. A. Wesson D. W. (2012). Parallel odor processing by two anatomically distinct olfactory bulb target structures. *PLoS One* 7:e34926. 10.1371/journal.pone.0034926 22496877 PMC3319618

[B65] Peng M. Coutts D. Wang T. Cakmak Y. O. (2019). Systematic review of olfactory shifts related to obesity. *Obes. Rev.* 20 325–338. 10.1111/obr.12800 30450791

[B66] Poessel M. Freiherr J. Wiencke K. Villringer A. Horstmann A. (2020). Insulin resistance is associated with reduced food odor sensitivity across a wide range of body weights. *Nutrients* 12:2201. 10.3390/nu12082201 32721994 PMC7468861

[B67] Proserpio C. de Graaf C. Laureati M. Pagliarini E. Boesveldt S. (2017). Impact of ambient odors on food intake, saliva production and appetite ratings. *Physiol. Behav.* 174 35–41. 10.1016/j.physbeh.2017.02.042 28259807

[B68] Ramaekers M. G. Boesveldt S. Gort G. Lakemond C. M. van Boekel M. A. Luning P. A. (2014). Sensory-specific appetite is affected by actively smelled food odors and remains stable over time in normal-weight women. *J. Nutr.* 144 1314–1319. 10.3945/jn.114.192567 24966408

[B69] Ramaekers M. G. Verhoef A. Gort G. Luning P. A. Boesveldt S. (2016). Metabolic and sensory influences on odor sensitivity in humans. *Chem. Senses* 41 163–168. 10.1093/chemse/bjv068 26567260

[B70] Reilly S. M. Saltiel A. R. (2017). Adapting to obesity with adipose tissue inflammation. *Nat. Rev. Endocrinol.* 13 633–643. 10.1038/nrendo.2017.90 28799554

[B71] Richardson B. E. Vander Woude E. A. Sudan R. Thompson J. S. Leopold D. A. (2004). Altered olfactory acuity in the morbidly obese. *Obes. Surg.* 14 967–969. 10.1381/0960892041719617 15329187

[B72] Riera C. E. Dillin A. (2016). Emerging role of sensory perception in aging and metabolism. *Trends Endocrinol. Metab.* 27 294–303. 10.1016/j.tem.2016.03.007 27067041

[B73] Rivière S. Soubeyre V. Jarriault D. Molinas A. Léger-Charnay E. Desmoulins L. (2016). High Fructose Diet inducing diabetes rapidly impacts olfactory epithelium and behavior in mice. *Sci. Rep.* 6:34011. 10.1038/srep34011 27659313 PMC5034277

[B74] Rombaux P. Duprez T. Hummel T. (2009). Olfactory bulb volume in the clinical assessment of olfactory dysfunction. *Rhinology* 47 3–9. 19382487

[B75] Rosenthal R. J. Morton J. Brethauer S. Mattar S. De Maria E. Benz J. K. (2017). Obesity in America. *Surg. Obes. Relat. Dis.* 13 1643–1650. 10.1016/j.soard.2017.08.002 28935198

[B76] Rottstädt F. Han P. Weidner K. Schellong J. Wolff-Stephan S. Strauß T. (2018). Reduced olfactory bulb volume in depression-A structural moderator analysis. *Hum. Brain Mapp.* 39 2573–2582. 10.1002/hbm.24024 29493048 PMC6866619

[B77] RStudio Team (2016). *RStudio: Integrated Development Environment for R.* Boston, MA: RStudio Team.

[B78] Russo C. Russo A. Pellitteri R. Stanzani S. (2018). Ghrelin-containing neurons in the olfactory bulb send collateralized projections into medial amygdaloid and arcuate hypothalamic nuclei: neuroanatomical study. *Exp. Brain Res.* 236 2223–2229. 10.1007/s00221-018-5298-z 29845448

[B79] Saad M. J. A. Santos A. Prada P. O. (2016). Linking gut microbiota and inflammation to obesity and insulin resistance. *Physiology* 31 283–293. 10.1152/physiol.00041.2015 27252163

[B80] Sato T. Homma R. Nagayama S. (2020). Direct comparison of odor responses of homologous glomeruli in the medial and lateral maps of the mouse olfactory bulb. *eNeuro* 7 10.1523/ENEURO.0449-19.2020 31974110 PMC7073388

[B81] Savic I. Gulyas B. Larsson M. Roland P. (2000). Olfactory functions are mediated by parallel and hierarchical processing. *Neuron* 26 735–745. 10.1016/S0896-6273(00)81209-X 10896168

[B82] Schriever V. A. Reither N. Gerber J. Iannilli E. Hummel T. (2013). Olfactory bulb volume in smokers. *Exp. Brain Res.* 225 153–157. 10.1007/s00221-012-3356-5 23212471

[B83] Skrandies W. Zschieschang R. (2015). Olfactory and gustatory functions and its relation to body weight. *Physiol. Behav.* 142 1–4. 10.1016/j.physbeh.2015.01.024 25619950

[B84] Stafford L. D. Whittle A. (2015). Obese individuals have higher preference and sensitivity to odor of chocolate. *Chem. Senses* 40 279–284. 10.1093/chemse/bjv007 25771359

[B85] Stevens G. A. Singh G. M. Lu Y. Danaei G. Lin J. K. Finucane M. M. (2012). National, regional, and global trends in adult overweight and obesity prevalences. *Popul. Health Metr.* 10:22. 10.1186/1478-7954-10-22 23167948 PMC3543235

[B86] Stunkard A. J. Messick S. (1985). The three-factor eating questionnaire to measure dietary restraint, disinhibition and hunger. *J. Psychosom. Res.* 29 71–83. 10.1016/0022-3999(85)90010-8 3981480

[B87] Thanarajah S. Hoffstall V. Rigoux L. Hanssen R. Brüning J. C. Tittgemeyer M. (2019). The role of insulin sensitivity and intranasally applied insulin on olfactory perception. *Sci. Rep.* 9:7222. 10.1038/s41598-019-43693-7 31076634 PMC6510903

[B88] Thiebaud N. Johnson M. C. Butler J. L. Bell G. A. Ferguson K. L. Fadool A. R. (2014). Hyperlipidemic diet causes loss of olfactory sensory neurons, reduces olfactory discrimination, and disrupts odor-reversal learning. *J. Neurosci.* 34 6970–6984. 10.1523/JNEUROSCI.3366-13.2014 24828650 PMC4019806

[B89] Thomann P. A. Dos Santos V. Toro P. Schönknecht P. Essig M. Schröder J. (2009). Reduced olfactory bulb and tract volume in early Alzheimer’s disease–A MRI study. *Neurobiol. Aging* 30 838–841. 10.1016/j.neurobiolaging.2007.08.001 17875348

[B90] Tong J. Mannea E. Aime P. Pfluger P. T. Yi C. X. Castaneda T. R. (2011). Ghrelin enhances olfactory sensitivity and exploratory sniffing in rodents and humans. *J. Neurosci.* 31 5841–5846. 10.1523/JNEUROSCI.5680-10.2011 21490225 PMC3089941

[B91] Tsai S.-Y. Gildengers A. G. Hsu J.-L. Chung K.-H. Chen P.-H. Huang Y.-J. (2019). Inflammation associated with volume reduction in the gray matter and hippocampus of older patients with bipolar disorder. *J. Affect. Disord.* 244 60–66. 10.1016/j.jad.2018.10.093 30317016

[B92] Turetsky B. I. Moberg P. J. Yousem D. M. Doty R. L. Arnold S. E. Gur R. E. (2000). Reduced olfactory bulb volume in patients with schizophrenia. *Am. J. Psychiatry* 157 828–830. 10.1176/appi.ajp.157.5.828 10784482

[B93] Weiss M. S. Hajnal A. Czaja K. Di Lorenzo P. M. (2019). Taste responses in the nucleus of the solitary tract of awake obese rats are blunted compared with those in lean rats. *Front. Integr. Neurosci.* 13:35. 10.3389/fnint.2019.00035 31417373 PMC6683675

[B94] Weiss T. Soroka T. Gorodisky L. Shushan S. Snitz K. Weissgross R. (2020). Human olfaction without apparent olfactory bulbs. *Neuron* 105 35–45.e5. 10.1016/j.neuron.2019.10.006 31706696 PMC6953431

