# Supplementary material for: Reduced Olfactory Bulb Volume in Obesity and Its Relation to Metabolic Health Status
Source: Front Hum Neurosci. 2020 Nov 27;14:586998. doi: 10.3389/fnhum.2020.586998 (PMC7729134; doi:10.3389/fnhum.2020.586998)
Supplement: Supplementary Table 1 — Correlation analysis of eating behavior questionnaires and BMI/OB volume. [file Table_1.docx]

Supplementary Material

# Supplementary Table 1

|  | Total | Normal weight | Overweight | Obese | p-value/F value between NW and OBE | Correlation with BMI | Correlation with OB volume |
| --- | --- | --- | --- | --- | --- | --- | --- |
|  | (n=67, 33 females) | (n=28, 14 females) | (n=11, 5 females) | (n=28, 14 females) | | p value/r | p value/r |
| TFEQ |  |  |  |  |  |  |  |
| Cognitive Restraint | 7.40±2.32 | 6.70±2.15 | 7.45±2.51 | 8.12±2.28 | **.027/5.209*^a^** | **.041/.258*^b^** | .379/-.114 ^b^ |
| Disinhibition | 6.41±2.45 | 6.07±2.04 | 6.55±2.16 | 6.72±2.98 | .325/.987 ^a^ | .359/.118 ^b^ | .799/-.033 ^b^ |
| Hunger | 5.30±2.31 | 4.78±1.87 | 5.64±2.62 | 5.72±2.56 | .117/2.548 ^a^ | **.049/.249* ^b^** | .475/-.092 ^b^ |
|  |  |  |  |  |  |  |  |
| DFS |  |  |  |  |  |  |  |
| Fat | 27.41±5.04 | 26.54±5.58 | 28.09±4.18 | 28.08±4.76 | .178/1.870 ^a^ | .273/.139 ^b^ | .855/.023 ^c^ |
| Free sugar | 10.66±3.41 | 10.25±3.74 | 9.82±3.09 | 11.48±3.11 | .162/2.016 ^a^ | .700/-.049 ^b^ | .434/.100 ^b^ |
| Fat and sugar | 19.02±4.68 | 19.04±4.06 | 19.64±3.50 | 18.72±5.78 | .830/.047 ^a^ | .891/-.017^b^ | .073/.227 ^c^ |
| Sumscore | 57.08±10.53 | 55.82±11.26 | 57.55±7.48 | 58.28±11.04 | .338/.936 ^a^ | .338/.122 ^b^ | .395/.109 ^c^ |
|  |  |  |  |  |  |  |  |
| FCQ |  |  |  |  |  |  |  |
| Lack of control | 22.77±7.28 | 21.54±6.84 | 21.45±7.37 | 24.72±7.57 | .080/3.197 ^a^ | .100/.213^b^ | .655/.057 ^b^ |
| Reinforcement | 20.10±6.37 | 20.92±6.93 | 18.36±5.46 | 20.00±6.21 | .759/.095 ^a^ | .674/.055 ^b^ | .883/-.019 ^b^ |
| Guilt | 19.57±6.71 | 18.42±6.97 | 19.00±5.33 | 21.08±6.95 | .083/3.134 ^a^ | .070/.234 ^b^ | .275/-.143 ^b^ |
| Emotions | 9.82±4.06 | 9.85±3.85 | 8.82±3.31 | 10.25±4.64 | .661/.195 ^a^ | .458/.097 ^b^ | .968/-.005 ^b^ |
| Cues | 13.84±3.62 | 13.73±3.39 | 14.09±4.78 | 13.83±3.42 | .812/.058 ^a^ | .774/.038 ^b^ | .255/.149 ^c^ |
| Hunger | 12.48±3.08 | 12.54±3.13 | 11.64±3.14 | 12.79±3.06 | .496/.471 ^a^ | .907/.015 ^b^ | .465/-.096 ^b^ |
| Sumscore | 98.57±25.34 | 97.00±24.50 | 93.36±25.44 | 102.67±26.61 | .280/1.194 ^a^ | .212/.162 ^b^ | .846/-.026 ^b^ |
|  |  |  |  |  |  |  |  |
| FCQ-C |  |  |  |  |  |  |  |
| Chocolate thoughts | 16.47±6.22 | 17.04±6.69 | 14.73±4.15 | 16.67±6.58 | .915/.011 ^a^ | .741/.044 ^b^ | .058/.248 ^b^ |
| Control | 14.74±5.70 | 13.88±4.89 | 13.18±4.40 | 16.37±6.73 | .124/2.455 ^a^ | .119/.203 ^b^ | .054/.252 ^b^ |
| Sumscore | 31.22±10.83 | 30.92±10.28 | 27.91±7.93 | 33.04±12.42 | .456/.565 ^a^ | .338/.126 ^b^ | .**033/.278* ^b^** |
|  |  |  |  |  |  |  |  |
| BIS-15 |  |  |  |  |  |  |  |
| Non-planning | 10.40±3.06 | 11.04±3.27 | 10.55±3.08 | 9.68±2.78 | .064/3.597 ^a^ | .095/-.214 ^b^ | .061/.241 ^b^ |
| motor | 10.87±2.57 | 11.04±2.52 | 10.82±1.94 | 10.72±2.92 | .649/.210 ^a^ | .898/-.017 ^b^ | .718/.047 ^b^ |
| attentional | 9.35±2.68 | 9.35±2.94 | 9.45±2.81 | 9.32±2.45 | .833/.045 ^a^ | .901/-.016 ^b^ | .442/.100 ^b^ |

TFEQ, Three factor eating questionnaire; DFS, Dietary fat and sugar intake questionnaire; FCQ, Food craving questionnaire; FCQ-C, chocolate craving questionnaire; BIS-15, Barrat impulsivity scale. ^a^ One-way analysis of variance (ANOVA) ^b^Spearman’s rank-order correlation for questionnaires and BMI and partial Spearman’s rank order correlation with the covariate sex for questionnaires and OB volume ^c^ Partial Pearson’s correlation with the covariate sex. * p ≤ 0.05; Bold font indicates statistical significance.
